# Micronutrient Levels and Supplement Intake in Pregnancy after Bariatric Surgery: A Prospective Cohort Study

**DOI:** 10.1371/journal.pone.0114192

**Published:** 2014-12-03

**Authors:** Roland Devlieger, Isabelle Guelinckx, Goele Jans, Willy Voets, Caroline Vanholsbeke, Greet Vansant

**Affiliations:** 1 Department of Obstetrics and Gynecology, University Hospitals Leuven, Leuven, Belgium; 2 Department of Development and Regeneration, KU Leuven, Leuven, Belgium; 3 Department of Public Health and Primary Care, KU Leuven, Leuven, Belgium; 4 Jessa Hospital, 3500 Hasselt, Belgium; 5 Hospital Oost-Limburg (ZOL), 3600 Genk, Belgium; Oslo University Hospital, Ullevål, Norway

## Abstract

**Background:**

Studies report frequent micronutrient deficiencies after bariatric surgery, but less is known about micronutrient levels of pregnant women after bariatric surgery.

**Objective:**

To prospectively evaluate micronutrient levels and supplement intake in pregnancy following bariatric surgery.

**Design:**

A multicenter prospective cohort study including women with restrictive or malabsorptive types of bariatric surgery. Nutritional deficiencies, together with supplement intake, were screened during pregnancy.

**Results:**

The total population included 18 women in the restrictive and 31 in the malabsorptive group. Most micronutrients were depleted and declined significantly during pregnancy. The proportion of women with low vitamin A and B-1 levels increased to respectively 58 and 17% at delivery (*P* = 0.005 and 0.002). The proportion of women with vitamin D deficiency decreased from 14% at trimester 1 to 6% at delivery (*P* = 0.030). Mild anemia was found in respectively 22 and 40% of the women at trimester 1 and delivery. In the first trimester, most women took a multivitamin (57.1%). In the second and third trimester, the majority took additional supplements (69.4 and 73.5%). No associations were found between supplement intake and micronutrient deficiencies.

**Conclusion:**

Pregnant women with bariatric surgery show frequent low micronutrient levels. Supplementation partially normalizes low levels of micronutrients.

## Introduction

Obesity is becoming more prevalent among women of reproductive age. A study by Bogaerts et al. (2012) reported that in 2009, one third of the women in the northern region of Belgium were overweight (21.6%) or obese (10.%) [1]. Caloric restrictions and increased exercise are the first-line treatment for obesity. However, repeated attempts often fail to induce long-term sustained weight loss. Therefore, the use of bariatric surgery as a therapy has markedly increased [2]. The surgery induces weight loss resulting in improved fertility and a reduced risk of obstetrical complications, including gestational diabetes, macrosomia and hypertensive disorders of pregnancy. However, the risk of complications such as intra-uterine growth restriction, nutritional deficiencies or intestinal hernias may surface [3], [4]. Nutritional deficiencies during pregnancy after bariatric surgery with serious consequences for the baby have been reported [3]. Although general recommendations exist, no specific prospective data is available on deficiencies throughout pregnancy and the effect of supplementation.

The objective of this study was to prospectively evaluate micronutrient levels during pregnancy and the effect of a standardized supplementation strategy in pregnant women who have undergone bariatric surgery.

## Subjects and Methods

### Design

A multicenter prospective cohort study was conducted from April 2009 until January 2011 at the antenatal clinics of Obstetrics and Gynecology departments in 5 hospitals in the Flemish part of Belgium. The study protocol was approved by the central Ethics Committee (Commission Medical Ethics University Hospitals KU Leuven/campus Gasthuisberg – S50984) and the local Ethics Committees (Commissions Ethics Committees Hospital Oost-Limburg Genk, Virga Jesse Hospital Hasselt, Sint-Augustinus Hospital Wilrijk, Imelda Hospital Bonheiden). All participants signed a written informed consent.

### Subjects

All pregnant women of West-European origin older than 18 years with a medical history of bariatric surgery presenting at the antenatal clinic before 15 weeks amenorrhea were eligible for recruitment. Exclusion criteria were multiple pregnancy, age less than 18 years and inclusion after 15 weeks pregnancy. Subjects were divided into 2 groups according to the type of surgery: a restrictive group and a malabsorptive group including the purely malabsorptive procedures as well as the mixed procedures.

### Clinical parameters

Following confirmation of eligibility and informed consent, patients' baseline characteristics were assessed and recorded. The baseline data focused on the type of surgery, the interval between surgery and conception, preoperative and preconceptional weight and height. During the first antenatal visit, all patients were recommended to use a standard prenatal multivitamin supplement. In case of observed micronutrient deficiencies during the first or second trimester and at delivery, the obstetrician provided a prescription for the required supplementation. **Table 1** gives an overview of the prescribed supplements for each observed micronutrient deficiency.

**Table 1:** Overview of the prescribed supplements for each micronutrient deficiency.

Folate	Folavit	1 x/day = 4 mg/day
Iron	Fero-Grad 500	1 x/day = 105 mg Fe2+
Vitamin B-12	Neurobion	1x/month = 500-1000 µg IM
Vitamin A	Omnibionta prenatal	1x/day = Vit C 180 mg, nicotinamide 20 mg, vit E 12 mg, vit B-5 10 mg, Provit A 3 mg, vit B-6 2.2 mg; vit B-2 1.6 mg, vit B-9 200 µg, vit B-8 100 µg, vit D 10 µg, vit B-12 2.7 µg, Mg 70 mg, Fe 28 mg, Zn 15 mg, Mn 1 mg, DHA 200 mg, vit E 12 mg
Vitamin D + Calcium	D-Vital forte	1 x/day = calciumcarbonate 2.5 g+, colecalciferol 880 IU, 1 g Ca2+
Vitamin B-1	Befacte Forte	1-3x/day
Multivitamin	Omnibionta prenatal	1x/day = Vit C 180 mg, nicotinamide 20 mg, vit E 12 mg, vit B-5 10 mg, Provit A 3 mg, vit B-6 2.2 mg; vit B-2 1.6 mg, vit B-9 200 µg, vit B-8 100 µg, vit D 10 µg, vit B-12 2.7 µg, Mg 70 mg, Fe 28 mg, Zn 15 mg, Mn 1 mg, DHA 200 mg, vit E 12 mg

Abbreviations: vitamin (vit), milligram (mg), gram (g), intramuscular (IM), microgram (µg), international unit (IU), Magnesium (Mg), Iron (Fe), Zinc (Zn), Manganese (Mn), Docosahexaenoic Acid (DHA)

Follow-up assessments were based on the Belgian standard clinical protocol for pregnant women [5], including a minimum of three ultrasound examinations with additional examinations if required, blood pressure measurement and urine screening for proteinuria and glucosuria at each antenatal visit. Furthermore, duration of pregnancy, presence of gestational diabetes, pregnancy-induced and chronic hypertension, induction of labor, mode of delivery, birth weight and height, macrosomia (birth weight ≥4000 g), low birth weight (birth weight <2.5 kg) and intrauterine growth restriction (weight for gestational age <P10), Apgar scores after 1 and 5 minutes and admission to intensive neonatal care were recorded. Gestational diabetes was diagnosed in accordance to the Carpenter-Coustan criteria using two or more abnormal plasma glucose values [6]. In case of severe dumping symptoms, assessment of the fasting glucose or glycosylated hemoglobin (HbA1c) was used as an alternative for the Oral Glucose Tolerance test. Pregnancy-induced hypertension was defined as follows: *de novo* blood pressure ≥140/90 mmHg appearing after 20 weeks of gestation. The definition of preeclampsia was the presence of pregnancy induced hypertension or chronic hypertension in combination with proteinuria [7]. Band management during pregnancy was based on the patients' symptomatology, and opened in case of vomiting or severe nausea after meals.

The dietary habits and the physical activity levels of the patients were recorded during the first and second trimester of pregnancy and have been reported elsewhere [8]. After the analysis, written lifestyle advice was offered to the patients. A healthy diet according to the official Belgian dietary guidelines was recommended [9], together with the refrain from alcohol and 30 minutes or more of moderate intensity physical activity on most, and preferably, all days of the week.

### Blood samples

A blood collection by venipuncture after an overnight fast was planned at 12 weeks of gestation. The screening contained a full hematology screening, as well as analyses for aspartate aminotransferase and alanine aminotransferase, ferritin, vitamin B-12, folate in red blood cells, albumin, hemoglobin and mean corpuscular volume. Blood collection was also analyzed for vitamin B-1 (thiamine), vitamin A (retinol), 25-OH-vitamin D (25-hydroxycholecalciferol) and vitamin E (α-tocopherol). The complete set of tests was repeated at 25 weeks of gestation and at the day of the delivery.

### Statistical analysis

All analyses were performed using the Statistical Package for the Social Sciences (SPSS for Windows, release 16.0). A two-sided level of significance of 0.05 was defined. Continuous variables of the two groups were compared using a Student's T test. If the assumption of normal distribution or equal variation was not fulfilled for one variable, a Mann Whitney U test was used. Categorical variables of the two groups were analyzed using a Chi Square Test or the Fishers' Exact Test. The evolution of proportion of deficiencies over the pregnancy trimesters was analyzed with the Cochrane's Q Test.

## Results

### Participants

Fifty four patients were recruited into the study of which 20 (37%) underwent a restrictive procedure and 34 (63%) a malabsorptive or mixed procedure. The restrictive procedures were all Laparoscopic Adjustable Gastric Bands (LAGB) and the malabsorptive procedures consisted of 33 Roux-en-Y Gastric Bypasses (RYGB) and one Biliopancreatic Diversion (BPD) or “Scopinaro”-procedure. The gastric band of seven patients in the restrictive group was deflated during pregnancy. Two patients dropped out of the study due to lack of interest and one twin pregnancy was excluded. In the restrictive group there was one termination of pregnancy following a prenatal diagnosis of neural tube defect. One patient in the malabsorptive group underwent a spontaneous miscarriage. The miscarriage rate in this study population was therefore 2% (1/54). As a result, the final analysis was performed on data from 49 women (91% of the originally recruited population).

### Baseline characteristics

The maternal characteristics are presented in **table 2**. Both groups were comparable for age at inclusion, height, mean preoperative weight and the time interval between the surgery and conception. 39% of the women were nullipara and 25% smoked at inclusion. The postoperative weight loss was significantly different between both groups, with the highest weight loss in the malabsorptive group. This resulted in a significantly lower prepregnancy weight and body mass index of the malabsorptive group compared to the restrictive group. The time between surgery and conception ranged from 2 months to 6.5 years. In the restrictive and malabsorptive group respectively 3 (17%) and 8 (26%), therefore 11 (22%) women in the total population, conceived within the first year after surgery (p = 0.724). All pregnancies, except for one, were planned. The woman who unexpectedly became pregnant was not using contraception and had fertility problems (anovulation) before surgery. She belonged to the malabsorptive group.

**Table 2:** Baseline characteristics of the study population according to type of bariatric surgery.

Baseline characteristic	Restrictive procedure(N = 18)	Malabsorptive procedure(N = 31)	P-value
Gestational age at inclusion (weeks)	9.5±2.2 (6–12)	10.7±2.5 (6-13)	0.075
Age (years)	30.4±3.2 (25-36)	29.6±5.5 (18-38)	0.506
Height (m)	1.7±0.1 (1.50–1.74)	1.7±0.1 (1.54–1.79)	0.838
Preoperative weight (kg)	108.6±13.5 (88–140)	116.0±19.9 (86–150)	0.205
Preoperative Body Mass Index (kg/m^2^)	40.0±4.9 (31–50)	42.4±6.4 (29–57)	0.178
Maximum postoperative weight loss (kg)	30.3±12.6 (16–57)	49.7±15.5 (22–80)	0.001
Interval between surgery and conception (months)	44.9±28.9 (4–108)	34.5±26.9 (2–96)	0.198
Prepregnancy weight (kg)	85.7±15.1 (63–110)	72.7±13.7 (54–112)	0.008
Prepregnancy Body Mass Index (kg/m^2^)	31.6±5.8 (22–44)	26.6±4.3 (22–39)	0.002
Nulliparae	6 (33)	13 (42)	0.551
Smokers	6 (33)	6 (19)	0.316

Values presented as mean ± SD (range) or n (valid percentage).

### Blood analysis

**Figure 1:**
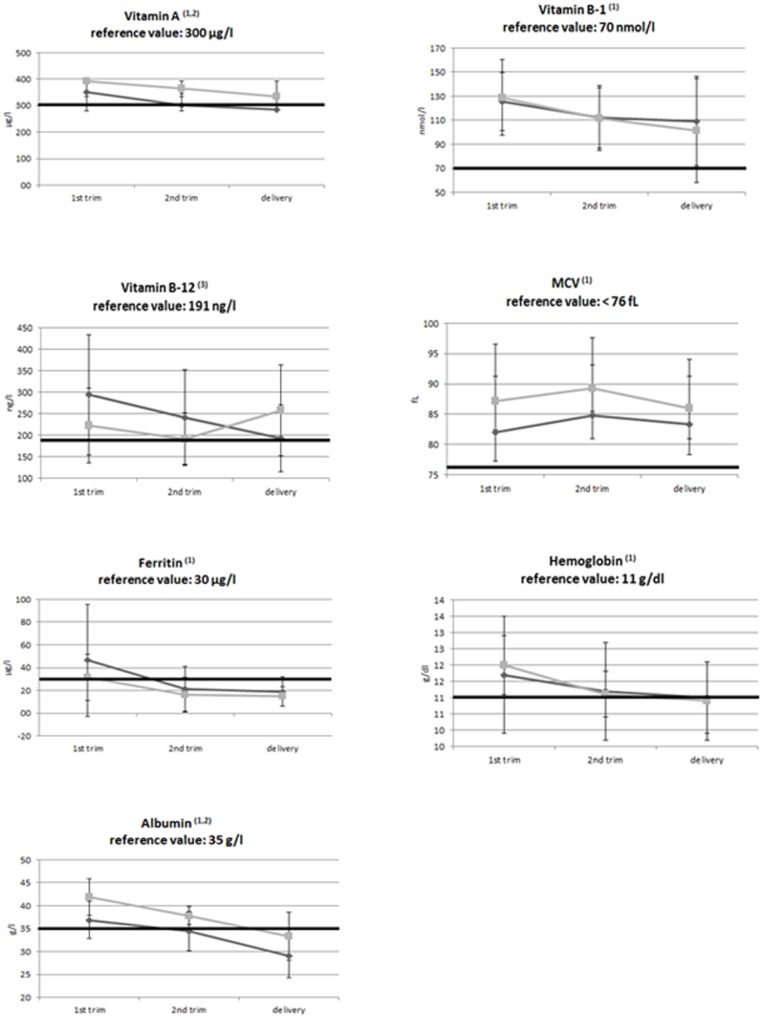
Mean (±SD) concentrations of various markers of nutritional/health status. light grey = malabsorptive group. dark grey = restrictive group. 1 = significant time effect. 2 = significant group effect. 3 = significant time x group effect.

**Figure 1** shows various markers of the nutritional health status. During pregnancy a decline in mean vitamin A concentration was observed (p = 0.037). The decline in vitamin A level was comparable in both groups, but the concentration in the restrictive group was consistently lower than in the malabsorptive group (p = 0.034). Another blood marker that declined significantly during pregnancy was vitamin B-1 (p = 0.018), without a difference between groups. A significant time by group interaction (p = 0.004) was noted for vitamin B-12: in the restrictive group the mean concentration decreased, whereas the mean concentration in the malabsorptive group increased. Mean corpuscular volume also changed: from the 1^st^ to the 2^nd^ trimester mean corpuscular cell volume increased whereas in the second half of pregnancy it decreased again (p = 0.017). Ferritin and hemoglobin levels declined significantly during pregnancy (p = 0.001 and p = 0.002 respectively). A last blood marker that declined during pregnancy was albumin (p = 0.001). The mean concentration of albumin also differed between groups with the lowest mean concentration in the restrictive group (p = 0.004) No significant changes during pregnancy, group difference or time by group interactions were observed for folic acid, alanine aminotransferase levels and for vitamin D and vitamin E levels.

**Table 3** shows the proportion of patients showing deficiencies across pregnancy and according to group. The proportion of patients with low serum levels of vitamin A increased significantly throughout pregnancy: 19% of women were deficient in the 1^st^ trimester, 40% in the 2^nd^ trimester and 58% at delivery (p = 0.005). Vitamin B-1 concentrations significantly declined during pregnancy and the proportion of vitamin B-1 deficiency increased throughout pregnancy (p = 0.002). The percentage of patients showing vitamin D deficiency significantly decreased due to effective supplementation (p = 0.030), but 36% of women had a 25-OH-vitamin level between 7–20 µg/l indicating a mild deficiency at the time of delivery. Another blood marker that declined significantly was albumin (p = 0.001), with 7% of patients being deficient in the first trimester, 21% in de second trimester and 74% at delivery. Based on low levels of ferritin, mean corpuscular cell volume and hemoglobin, the diagnosis of mild anemia (hemoglobin <11 g/dl) was made in 22% of the women during the 1^st^ trimester and 40% of the women at delivery. For each trimester, we found no differences in the proportion of women with one or more micronutrient deficiencies when comparing the group of women conceiving within the first year of surgery and the women conceiving after the first year of surgery (1^st^ trimester: 81.8 versus 81.6%, *P* = 0.986; 2^nd^ trimester: 90.9 versus 92.1%, *P* = 0.898 and delivery: 81.8 versus 89.5%, *P* = 0.495).

**Table 3:** Proportion of deficiencies (%) across pregnancy and according to group.

	Total population (N = 49)	Restrictive procedure(N = 18)	Malabsorptive procedure(N = 31)	p-value group
Albumin <35 g/l				
1^st^ trimester	7	15	3	0.165
2^nd^ trimester	21	33	15	0.221
3^rd^ trimester	74	91	65	0.115
P-value trimester	0.001	0.011	0.001	
Hemoglobin <11 g/dl				
1^st^ trimester	28	29	28	0.946
2^nd^ trimester	63	44	73	0.048
3^rd^ trimester	76	71	79	0.397
P-value trimester	0.001	0.247	0.001	
MCV <76 fL				
1^st^ trimester	12	29	3	0.016
2^nd^ trimester	9	13	7	0.459
3^rd^ trimester	26	41	14	0.060
P-value trimester	0.156	0.717	0.174	
Vitamin B-12 <191 ng/l				
1^st^ trimester	35	31	37	0.709
2^nd^ trimester	45	39	48	0.564
3^rd^ trimester	41	58	32	0.281
P-value trimester	0.735	0.156	0.311	
Ferritin <30 µg/l				
1^st^ trimester	27	14	33	0.186
2^nd^ trimester	54	36	63	0.097
3^rd^ trimester	42	55	36	0.319
P-value trimester	0.076	0.174	0.093	
Folic Acid <252 µg/l				
1^st^ trimester	3	0	4	0.698
2^nd^ trimester	0	0	0	0.693
3^rd^ trimester	0	0	0	0.379
P-value trimester	0.202	0.368	0.236	
Vitamin B-1 <70 nmol/l				
1^st^ trimester	2	0	4	0.558
2^nd^ trimester	5	0	7	0.235
3^rd^ trimester	17	6	26	0.257
P-value trimester	0.002	0.135	0.011	
Vitamin A <300 µg/l				
1^st^ trimester	19	18	19	0.885
2^nd^ trimester	40	56	31	0.098
3^rd^ trimester	58	56	59	0.861
P-value trimester	0.005	0.205	0.013	
25-OH-Vitamin D <7 µg/l				
1^st^ trimester	14	12	16	0.538
2^nd^ trimester	7	13	4	0.499
3^rd^ trimester	6	12	0	0.199
P-value trimester	0.030	0.368	0.135	
25-OH-Vitamin D 7–20 µg/l				
1^st^ trimester	26	35	20	0.538
2^nd^ trimester	21	19	23	0.499
3^rd^ trimester	36	29	42	0.199
P-value trimester	0.368	0.565	0.197	
Vitamin E <5.0 mg/l				
1^st^ trimester	2	0	3	0.447
2^nd^ trimester	0	0	0	0.662
3^rd^ trimester	0	0	0	0.235
P-value trimester	0.368	0.607	0.368	

Values presented as proportion (%).

**Table 4** summarizes the association between supplement intake and micronutrient deficiency across the different pregnancy trimesters. The majority of women who had a micronutrient deficiency in the first pregnancy trimester reported to already take a supplement at that time (n = 37/40, 92.5%, *P* = 0.380). Of the 45 women with a micronutrient deficiency in the second pregnancy trimester, and with corresponding data on self-reported supplement intake in the first trimester, 41 women (n = 41/45, 91.1%) was taken a supplement in trimester 1 of pregnancy (*P* = 0.273). No association was found between micronutrient deficiency in trimester 2 and supplement intake in that same period (*P* = 0.377). Also no associations were seen between micronutrient deficiencies at birth and self-reported supplement intake during trimester 1 and 2 of pregnancy (*P* = 0.668 and *P* = 0.839) and at birth (*P* = 0.545). Again, the majority of women with a micronutrient deficiency at birth reported to take a supplement during trimester 1 (n = 38/43, 88.4%), trimester 2 (n = 38/41, 92.6%) and around the moment of birth itself (n = 33/41, 80.5%).

**Table 4:** Association between supplement intake and micronutrient deficiency across the different pregnancy trimesters.

	Reported vitamin/mineral supplement			
Micronutrient deficiency	No	Multivitamin/mineral	Additional supplement	P-value
Trimester 1	Trimester 1			0.380
Yes (n = 40)	3 (7.5)	23 (57.5)	14 (35.0)	
No (n = 9)	2 (22.2)	5 (35.6)	2 (22.2)	
Trimester 2	Trimester 1			0.273
Yes (n = 45)	4 (8.9)	25 (55.6)	16 (35.6)	
No (n = 4)	1 (25.0)	3 (75.0)	0	
	Trimester 2			0.377
Yes (n = 43)	2 (4.4)	8 (17.8)	33 (73.3)	
No (n = 4)	1 (25.0)	1 (25.0)	2 (50.0)	
Trimester 3	Trimester 1			0.668
Yes (n = 43)	5 (11.6)	24 (55.8)	14 (32.6)	
No (n = 6)	0	4 (66.7)	2 (33.3)	
	Trimester 2			0.839
Yes (n = 41)	3 (7.0)	8 (18.6)	30 (69.8)	
No (n = 6)	0	1 (16.7)	5 (83.8)	
	Trimester 3			0.545
Yes (n = 41)	8 (18.6)	3 (7.0)	30 (73.2)	
No (n = 6)	0	1 (16.7)	5 (83.3)	

Values presented as n (valid percentage).

During the first trimester, almost 90% of the pregnant women reported to take a supplement (n = 44). A multivitamin was taken by 28 (57.1%) women and an iron supplement by 10 women (20.4%). During the second trimester, multivitamins were taken by 10 (20.4%) women. Fourteen women (29.1%) took a vitamin B-12 supplement and 6 (12.5%) women an iron supplement. At delivery, a multivitamin, an iron supplement and a vitamin B-12 supplement were taken by respectively 4 (8.2%), 24 (50.0%) and 11 (22.9) women.

### Maternal and neonatal outcomes

There was one case of pre-eclampsia and one of woman was diagnosed with gestational diabetes in the malabsorptive group. Two patients were diagnosed with PIH in the restrictive group and 3 in the malabsorptive group. Mean birth weight was significantly lower in the malabsorptive group (3.06±0.56) compared to the restrictive group (3.43±0.47) (p = 0.024). There was one preterm birth (<37 weeks of gestation) in the restrictive group and 4 in the malabsorptive group (p = 0.639). No low birth weight was detected in the restrictive and 2 in the malabsorptive group (p = 0.526). Besides the miscarriage and the termination of pregnancy due to the diagnosis of neural tube defect mentioned earlier, two more relevant adverse events occurred. Two patients of the malabsorptive group were diagnosed with a small bowel obstruction (**table 5**).

**Table 5:** Maternal and neonatal outcomes.

	Restrictive procedure (N = 18)	Malabsorptive procedure (N = 31)	P-value
Pregnancy duration (weeks)	39.28±1.40 (36–42)	38.31±2.15 (29–41)	0.094
Gestational weight gain (kg)	13.06±8.05 (0–30)	12.77±6.06 (2–23)	0.892
Birth weight (kg)	3.43±0.47 (2.62–4.16)	3.06±0.56 (0.95–4.13)	0.024
Infant length (cm)	49.94±1.67 (47–53)	49.15±1.66 (46–53)	0.092
Gestational diabetes	1 (7.7%)	1 (5.6%)	1.000
Pregnancy induced hypertension	2 (11%)	3 (10%)	1.000
Chronic hypertension	0	0	-
Preeclampsia	0	1 (3%)	1.000
Induction of labor	3(17%)	3 (10%)	0.798
Caesarean section	3 (17%)	9 (30%)	0.624
Preterm birth <37 weeks	1 (6%)	4(13%)	0.639
Low birth weight (<2.5 kg)	0 (0%)	2 (7%)	0.526
Macrosomia (> 4.0 kg)	3 (17%)	1 (3%)	0.134
Shoulder dystocia	0	0	-
Low Apgar score at 1 min (<4)	0	1 (3%)	0.311
Low Apgar score at 5 min (<4)	0	0	-
Admission at neonatal intensive care	1 (6%)	3 (10%)	0.587

Values presented as n (valid percentage).

## Discussion

We show that women undertaking pregnancy after bariatric surgery are at high risk for low micronutrient levels, especially for vitamins A and B-1 and albumin. We document these deficiencies during each trimester of pregnancy. Substitution only partially normalizes these low levels. In contrast to expectations, purely restrictive types of surgery are also associated with nutritional deficiencies in a significant proportion of patients.

Our results need to be carefully interpreted in the light of both the physiological pregnancy changes in absorption and metabolism, as well as the specific alternations due to obesity surgery. Pregnancy is an anabolic and dynamic state where hormones redirect nutrients to maternal tissues and to the developing fetus. The demand for both energy and micronutrients is increased and the concentration of circulating nutrient-binding proteins and micronutrients is decreased. An altered maternal kidney causes an increased urine excretion of water-soluble vitamins takes place [10]. Maternal fat storage increases in early to mid-gestation, which may affect the level of fat-soluble vitamin [11]. Finally, shifts in plasma volumes can result in decreased biomedical indexes for minerals and trace elements [10]. After bariatric surgery, further changes are observed. In the restrictive procedures, a gastric pouch is created by the use of staples or a band. Although the intestinal continuity is left intact with a normal digestion and absorption of nutrients, deficiency of micronutrients can occur because of several reasons: a restricted food intake, poor eating behavior, low nutrient-dense food choices and food intolerance [12], [13]. The malabsorptive procedures bypass segments of the gastrointestinal tract (duodenum and jejunum) and limit the amount of intestine available for absorption [13]. The mixed procedures limit food intake by creating a gastric pouch combined with a limited amount of intestine available for absorption. Less gastric acid, bile and pancreatic enzymes are available for the absorption of nutrients [13].

Most studies outside pregnancy found vitamin deficiencies to be more common in the malabsorptive and mixed procedures than in the purely restrictive procedures [3], [12]–[15]. In the current study, both the restrictive and the malabsorptive group showed comparable low micronutrient levels, although deficiencies were globally more pronounced and frequent in the malabsorptive group. Reflecting this, we found no significant differences in prescribed supplements between the two groups. Besides the relatively small studied population, this probably also reflects the specific changes in requirements, absorption and metabolism required to pregnancy. Persisting severe nausea and vomiting, which led to band deflation in a significant proportion of our patients, is known to be associated with important micronutrient deficiencies [16].

Fat-soluble vitamin deficiencies among bariatric surgery patients can occur because of a changed fat digestion that alters the digestion, absorption and transport of fat-soluble vitamins [12]. We found a decline in mean vitamin A concentrations, resulting in an increased proportion of pregnant women showing vitamin A deficiency. Besides the effect of surgery, vitamin A concentrations decline gradually in pregnancy because of hemodilution [17]. Vitamin D deficiency was frequent, but decreased during pregnancy following substitution of the patients with levels below 20 µg/l. Vitamin D deficiencies arise more frequently post-bariatric surgery because the intake of fortified dairy products is limited due to dietary intolerance and the bypassed primary absorption sites of calcium and vitamin D [18]. In normal pregnancy, the active form of vitamin D (1.25-dihydroxycholecalciferol) increases two to threefold during the first trimester whereas the inactive form (25-hydroxycholecalciferol) decreases [10].

Water-soluble vitamin deficiencies in non-pregnant subjects are more common after malabsorptive procedures than purely restrictive procedures [14]. In contrast to this, we found a decreased concentration of vitamin B-12 in the restrictive group. The mean concentration in the malabsorptive group also decreased from the first to the second trimester, but then increased during the second trimester and in late pregnancy. This was possibly due to more frequent supplementation with vitamin B-12 in the malabsorptive group during the second trimester and at delivery. Possible causes of vitamin B-12 deficiencies after surgery include limited intake of animal proteins and decreased gastric secretions that affect cleavage of vitamin B-12 from protein. Inadequate secretion and function of intrinsic factor can decrease absorption and bacterial overgrowth in the defunctionalized ileal segment. In pregnancy a progressive decline of serum vitamin B-12 concentrations occurs [18]. Only 3 patients in the malabsorptive group showed folate deficiencies during the first trimester. Folate deficiency can occur in pregnancy because of a decreased intestinal absorption, which is also affected in bariatric patients by bypassing the primary site of absorption, a decreased food intake and deficiency of vitamin B-12 which is a co-enzym to convert folate to its active form [10]. Our findings can be explained by the adequate intake of folic acid supplement as part of the multivitamin supplement prescribed in the first trimester to most of the women. The proportion of patients with low vitamin B-1 levels increased. Contributing factors to vitamin B-1 deficiency after surgery are persistent vomiting, inadequate dietary intake and rapid weight loss. In normal pregnancy, vitamin B-1 levels decrease [10].

Concentrations of hemoglobin, ferritin and mean corpuscular volume lower during normal pregnancy because of hemodilution [18]. Bariatric patients have a decreased production of hydrochloric acid in the stomach, which is necessary to convert dietary iron in the ferric form into the ferrous state. The reduced intake of meat and the reduction of intestinal iron absorbing capacity due to bypassing are other contributors of the often observed iron deficiency [19]. We found mild anemia in 22% of the women in the first trimester of pregnancy and in 40% of the women at delivery. Nomura et al. (2011) more specifically investigated the influence of time to conception interval of anemia in women with silastic RYGB. They found that iron deficiency anemia being more prevalent in women conceiving more than 4 years after surgery compared to women conceiving within 4 years after delivery. In both groups of women, the diagnosis of anemia was frequent (trimester 1: 53.3%, trimester 2: 76.7%, trimester 3: 60.0%) [20]. Another blood marker which declined significantly was albumin with the lowest mean concentration being observed in the restrictive group. Serum albumin concentration is frequently used as a global marker of nutritional status.

The potential harm of vitamin deficiencies is well documented [3], [21]. Low levels of micronutrients could only been covered partially in our study. We suspect that low compliance to the prescribed supplements may certainly be involved. Further research is needed to develop an effective approach for supplementation of this population at risk. Screening and supplementation for micronutrient deficiencies would optimally be studied in a placebo-controlled context. However, such an approach is ethically unacceptable in view of the known deleterious effects of vitamin deficiencies on the fetus. Providing these women with oral substitution may not be the best solution as absorption of medication in general, but more specifically iron and fat soluble vitamins, may be hampered through the surgery. Finally, an improvement of nutrition in this population could also be effective in decreasing the amount of nutritional deficiencies. We recently showed that nutritional intake is poor in this population [8].

Limitations of this study include a missing control group and the relatively small cohort size. However, a big effort is needed to prospectively evaluate a large sample of pregnant women after bariatric surgery and so far, this is the largest prospective series with evaluation during each trimester of pregnancy. Data on the reported baseline supplement intake are missing. During the first prenatal visit an extensive medical history was performed including a question related to the baseline intake of medication and supplements. For each patient recruited into the study, the surgeon who performed the bariatric surgery was contacted and requested to send a report of the surgery as well as the medication and supplements prescribed to the patient. However, as the period between the last visit with the surgeon and/or general practitioner and the first prenatal visit varied widely, these records were not considered to be reliable to reflect the actual baseline use of any medication or nutritional supplement. We used non-pregnant reference values, as there are no reference values for pregnancy available. Finally, no clear view is possible on the actual intake of supplements, while the compliance of these patients is known to be low [22].

In summary, this study shows that women who underwent bariatric surgery show frequent low micronutrients levels that could be potential harmful to themselves and their offspring. This information is of relevance to all clinicians involved in the care of this growing group of patients.
